# The Play at West Norfolk and Lynn Hospital

**Published:** 1913-01-04

**Authors:** 


### THE PLAY AT WEST NORFOLK AND LYNN HOSPITAL.

If this is our last Christmas before the National Insurance Act comes into force, we have had a very jolly one. Very hard we worked with our ward decorations, many hands helping, and in addition to the usual holly and ivy we had a scheme of decoration for each ward in paper flowers. One of the most effective proved to be the scarlet japonica, large thorn branches with buds of scarlet paper wired on the thorns; another ward chose sunflowers, and most natural-looking bulrushes; another arum lilies and poppies; trellised arches in the doorways covered with ramblers, and the children's ward in dainty pink and white, fairy hoops and silver bells, and pink and silver balls, with snow-flakes cunningly devised in white wool and string. We put up the big tree on Christmas Eve, and feasted our eyes on it till Saturday.

On Christmas Day all patients under sixteen found stockings on their bed-rails. Every patient had a parcel of clothes—shirts, socks, or petticoats. After a short service we assembled all who could be moved into a men's ward for dinner. The table decorations were pink and white chrysanthemums and trails of smilax, with sweets and crackers and wonderful candlesticks of plaster of Paris and glittering snow. Dinner was a joyous meal, especially when the plum-puddings came and the spinsters got rings and the bachelors thimbles; and there were miraculous escapes when buttons and pigs and coins were nearly swallowed.

Among the most lively patients were a Dane and a Dutchman, neither speaking English, but managing to understand the jokes. Each patient may ask a visitor to tea on Christmas Day, and we have a big tea in one of the women's wards for all who can come—nurses and servants, sisters, matron, and house surgeon; indeed, it is nearly as gay a meal as dinner. In the midst of the mirth a little girl of six began to weep bitterly, refused to explain why until her own nurse was fetched and inquiries in anxious whispers at last drew the reply: "My mother hasn't got a plate." Dolly sobbed this out, and not till her mother was speedily provided with one was all well.

Then came the great event of the day, the concert and a play. A real platform, with proper scenery, curtains, and footlights. Patients were carried in chairs and on stretchers, comfortably settled upon beds and sofas, their visitors round them, prepared to enjoy themselves enormously. It was such exquisite fun to see their nurses and the dignified sisters doing "Ten little nigger boys" in smocks and neckerchiefs—made from Linen Guild dusters of a loud check, "The Quaker Maid," "My Motter," "Yorkshire," all in character; and three Irish girls danced a three-hand reel. Then the farce, "Jerramy's Courtship," the house surgeon being the fire-eating captain, with sword and pistols, and the red-haired linendraper one of the honorary staff. To see him shaken by two of the pros, to see him escape up the chimney, and come down again all sooty and kiss a white-clad girl and leave black marks on her sleeves—all this caused delirious laughing and shouting. Even the most convinced pessimist would rock in sympathy with the hilarity around him. After "The King," we put our people to bed, tired but happy. One woman of forty said she had never seen a play before! All the mysteries of wigs and costumes and make-up had to be explained to her. On Saturday we had our Christmas tree, gifts for all in the hospital, including scrubbers and the errand-boy; a fine tree lighted with candles in the old way; Father Christmas in his traditional robes of scarlet and ermine, a flowing beard, and white hair. Greeted with cheers, he made a tour of the ward to welcome all, and then distributed his gifts amidst much noise of trumpets, whistles, drums, and organs. How the children's eyes sparkled and how they laughed and shouted at his jokes, some of them making a dainty curtsey to him. Presently a Witch flew in on a broomstick, causing a momentary silence and somewhat scaring Dolly, the afore-mentioned little girl; but a handsome little Spanish-looking baby of three shouted "It's Sister!"; and they all clapped her in her brave attire, crowned by a black beaver cone-shaped hat. The Witch alighted by a huge cake, iced in white and pink and mauve, and covered with mice and birds and fearsome things, but when the top was cut, behold, it was stuffed with parcels for everyone. Much merriment was caused by masks and faces sent by Her Majesty the Queen, and which were promptly worn by the recipients. Finally, Father Christmas and the Witch invited all to tea in the board-room.

